# Prophylactic Protection Against Salmonella typhimurium Infection by Single-Atom Zinc Catalysts

**DOI:** 10.3390/nano16090562

**Published:** 2026-05-02

**Authors:** Ling Teng, Hesheng Pan, Zhongwei Chen, Junfeng Sun, Yanwen Zhang, Changting Li, Zhe Pei, Chunxia Ma, Yu Gong, Huili Bai, Leping Wang, Yan Huang, Jing Wang, Chao Zhao, Xian Li, Yangyan Yin, Yingyi Wei, Hao Peng

**Affiliations:** 1 Guangxi Key Laboratory of Animal Breeding, Disease Control and Prevention, College of Animal Science and Technology, Guangxi University, Nanning 530004, Chinapanhesheng@163.com (H.P.);; 2 Key Laboratory of China (Guangxi)-ASEAN Cross-Border Animal Disease Prevention and Control, Ministry of Agriculture and Rural Affairs of China, Nanning 530001, China; 3 Guangxi Key Laboratory of Veterinary Biotechnology, Guangxi Veterinary Research Institute, Nanning 530001, China; 4 College of Food and Quality Engineering, Nanning University, Nanning 530299, China; 5 Virginia Tech, Blacksburg, VA 24060, USA; 6 Guizhou Provincial Livestock and Poultry Genetic Resources Management Station, Guiyang 550001, China; 7 College of Animal Science and Technology, West Campus, Guangxi Agricultural Engineering Vocational Technical College, Nanning 530004, China

**Keywords:** single-atom catalyst (SAC), antibacterial effect, intestinal flora, anti-*S. typhimurium* infection, international health

## Abstract

Zinc oxide promotes poultry growth, but it tends to agglomerate. This necessitates high doses and leads to environmental contamination from unabsorbed, excreted zinc. Undigested zinc is excreted and can enter the food chain, increasing the probability of zinc residues in edible poultry tissues (muscle, liver, and eggs) and raising concerns for consumer safety. MOF-supported single-atom zinc catalysts (SAC) resolve agglomeration by atomic anchoring, enhancing bioavailability. High-temperature/high-pressure fixation of Zn^2+^ surfaces was confirmed by XRD, while FESEM revealed the corresponding surface morphology, collectively verifying SAC formation. SAC exhibited potent antimicrobial efficacy against key pathogens such as *Salmonella typhimurium*, *Escherichia coli*, and *Staphylococcus aureus* (MIC of 3.125 mg/mL, MBC of 25 mg/mL). Co-culture experiments further demonstrated that the antibacterial performance of SAC remained stable over a temperature range of 20–80 °C and a pH range of 2–8, thus exhibiting excellent thermal stability and gastrointestinal tolerance. In 7-day-old chicks, SAC alleviated *S. typhimurium*-induced inflammation, reduced bacterial adherence, upregulated claudin-1, preserved gut homeostasis, ameliorated tissue lesions, and increased the abundance of *Lactobacillus* in the cecum, demonstrating promising potential for poultry infection control.

## 1. Introduction

The WHO indicates that foodborne diseases result in 600 million annual cases of food poisoning and 420,000 deaths, constituting a substantial burden on global public health (WHO, https://www.who.int/news-room/fact-sheets/detail/food-safety, URL (accessed on 14 December 2025)). Among foodborne pathogens, Salmonella typhimurium (*S. typhimurium*) is a primary threat to food safety and public health in developing nations due to its broad host range (encompassing humans, poultry, etc.) and high pathogenicity—while it typically causes self-limiting gastroenteritis in immunocompetent hosts, it may induce systemic complications or mortality in immunocompromised populations or specialized hosts [1].

Poultry products are identified as critical vectors for *Salmonella* contamination, and their systematic dissemination along the food safety chain follows a “source–initiation, diffusion–amplification, processing–dissemination, exposure–outbreak” framework [2]. Source–initiation lays the foundation for contamination: Salmonella in chicken feces, litter, and wastewater invade poultry via the respiratory tract, the digestive tract, or skin wounds, whereas contaminated feed or water directly infects poultry [3]. A representative example is the Class I recall by the U.S. FDA in 2024: 5.4 million eggs contaminated with *Salmonella* due to feed contamination resulted in 93 infections across three states (34 hospitalizations) (FDA, https://www.fda.gov/food/outbreaks-foodborne-illness/outbreak-investigation-salmonella-eggs-sept-2024, URL (accessed on 18 December 2025)), which underscores the critical importance of feed safety management at the breeding stage.

Previously, antibiotics were widely used in poultry production to inhibit colonization and proliferation of pathogenic bacteria; however, their overuse has driven the emergence of widespread drug resistance, complicating both prevention and treatment and promoting horizontal transfer of resistance genes among pathogens [4,5]. The emergence and dissemination of multidrug-resistant *S. typhimurium* strains represent a major public health challenge, thereby highlighting the need to develop antibiotic alternatives.

Zinc oxide helps repair the intestinal lining and stops pathogens from sticking by working through both astringent and antimicrobial mechanisms [6]. With advances in nanotechnology, researchers are now looking at zinc oxide nanoparticles (ZnO-NPs) as a new type of feed additive. However, conventional zinc oxide and ZnO-NPs both have poor bioavailability. In the gut environment, these particles tend to clump together, leading to uneven release of zinc ions and quick removal by the host [7]. Also, when high concentrations of zinc enter the environment via animal excreta (e.g., feces and urine), it disrupts the microbial community equilibrium and exerts adverse effects on other organisms within the ecosystem. It is noteworthy that ZnO-NPs and zinc oxide have oxidative stress-induced toxicity effects, and the toxicity of zinc oxide (ZnO) and ZnO nanoparticles is caused by their dissolution and subsequent release of toxic Zn^2+^ ions into aqueous medium. The dissolution of Zn^2+^ ions depends on the particle size, and the dissolution amount increases with an increase in particle size [8,9,10].

To address these challenges, single-atom catalysis (SAC) technology presents a promising solution by anchoring isolated Zn atoms onto metal–organic frameworks (MOFs), achieving near-100% atomic utilization efficiency and enhanced stability [11,12]. Single-atom zinc is fixed in the support through coordination (such as Zn^2+^ coordinated with nitrogen atoms in Zn-N-C catalysts), its state is stable and will not dissolve easily, and the lower rates of ion leaching can greatly reduce the toxic effects mediated by Zn^2+^ by inducing oxidative stress [13]. Previous studies by the same research team have reported the synthesis of novel single-atom catalysts and related works in multiple prestigious international journals, including *Journal of the American Chemical Society*, *Nano Research* and *ACS Catalysis* [2,14]. Single-atom catalysts feature well-defined and uniform active sites, which exhibit high activity and selectivity. Superior antibacterial properties are demonstrated by these catalysts. SAC have been shown to exhibit efficient catalytic activity in generating ROS in bacterial infection microenvironments with insufficient H_2_O_2_ concentrations [15,16]. This process has been demonstrated to induce oxidative stress responses in bacteria, leading to effective bacterial elimination while mitigating the risk of antibiotic resistance. These catalysts are structurally robust, recyclable, and environmentally benign, and are devoid of adverse effects on biological systems. Although SAC have been shown to exhibit superior antimicrobial activity against multidrug-resistant bacterial pathogens in vitro, they in vivo therapeutic potential, particularly in addressing systemic infections and modulating host–microbiota crosstalk, remains largely uncharacterized.

Based on past research, the following hypotheses have been proposed: SAC may have multiple protective effects against *Salmonella* infection in chickens—(i) *Salmonella* growth in chickens is inhibited by bactericidal activity; (ii) modulation of the gut microenvironment may increase resistance to *Salmonella* in chickens. To test these hypotheses, we evaluated the in vivo efficacy of SAC using a model of three yellow chickens and further explored its potential as a new supplement in health management.

## 2. Materials and Methods

### 2.1. Reagents

ZnO was purchased from Guangzhou Tao Sheng Chemical Industry Co., Ltd., Guangzhou, China. The RNA pure Tissue & Cell Kit (DNase I), HiFiScript cDNA Synthesis Kit and UltraSYBR Mixture were purchased from Beijing ComWin Biotech Co., Ltd., Beijing, China.

### 2.2. Preparation of SAC

**Figure 1:**
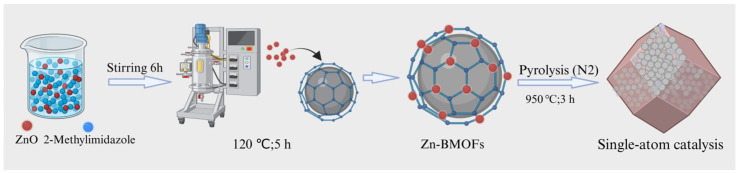
The preparation of single-atom zinc catalysts (image created in https://biogdp.com).

As illustrated in Figure 1, BMOFs were synthesized by stirring a methanol solution containing zinc oxide and dimethylimidazole. The resulting solution was transferred into a stainless-steel autoclave and sealed at 120 °C for 5 h. The product was collected by centrifugation, followed by washing with N,N-dimethylformamide until the supernatant became colorless. The obtained sample was then washed three times with methanol and dried under vacuum at 60 °C overnight. Finally, Zn-NCs were obtained by pyrolyzing the BMOF precursor at 950 °C for 3 h (with a heating rate of 3 K min^−1^) under a nitrogen atmosphere.

### 2.3. X-Ray Diffraction

The crystalline structures of ZnO and SAC were characterized by XRD. Following drying and pulverization, the samples were analyzed using a Rigaku SmartLab SE diffractometer (Rigaku Corporation, Tokyo, Japan).

### 2.4. FESEM and EDX Mapping

The morphology of single-atom catalysts was characterized using field-emission scanning electron microscopy (FESEM, Hitachi High-Technologies Corporation, Tokyo, Japan). The elemental composition of nanoparticles was determined by EDX mapping, which was collected using an integrated EDX detector (Bruker Nano GmbH, Berlin, Germany) equipped on the scanning electron microscope.

### 2.5. XPS

Samples were pressed into pellets and mounted on a sample holder, and then loaded into the sample chamber of a Thermo Scientific K-Alpha XPS spectrometer (Thermo Fisher Scientific, Waltham, MA, USA). The chamber was evacuated to below 2.0 × 10^−7^ mbar before transferring the sample to the analysis chamber. Measurements used a 400 μm spot size, 12 kV accelerating voltage, and 6 mA filament current. Survey scans were collected at 150 eV pass energy with 1 eV step size. High-resolution scans were collected at 50 eV pass energy with 0.1 eV step size.

### 2.6. EXAFS

Transmission XAS measurements were performed on a laboratory device (easyXAFS300, easyXAFS LLC, Spokane, WA, USA), which is based on Rowland circle geometries with spherically bent crystal analyzers (SBCAs, (Ge(331), Luxium Solutions, Hiram, Ohio, OH, USA)) and operated using a Ag X-ray tube source and a silicon drift detector (AXAS-M1, KETEK GmbH, Berlin, Germany).

### 2.7. Bacterial Strains

*Escherichia coli* (ATCC25922) and *Staphylococcus aureus* (ATCC6583) were both procured from the China Veterinary Microorganism Strain Preservation and Management Center. *Salmonella enterica serovar Typhimurium* (SM022) labeled with FITC was offered by Professor Alejandro Aballay at Duke University, USA.

### 2.8. Animals

A total of 200 one-day-old Sanhuang chickens were procured from Guangxi Jinling Husbandry Group Co., Ltd., Nanning, Guangxi Zhuang Autonomous Region, China. All animal experiments conformed to the Guide for the Care and Use of Laboratory Animals published by the US National Institutes of Health (NIH Publication, Eighth edition, 2016) and were approved by the Animal Welfare and Ethical Committee of Guangxi Veterinary Research Institute.

### 2.9. MIC and MBC

SAC and ZnO were separately suspended in LB broth to final concentrations of 100, 80, 50, 40, 25, 20, 12.5, 6.25, 3.125, and 1.5625 mg/mL. Subsequently, 1 mL of separate pathogen suspensions (*S. typhimurium*, *E. coli*, or *S. aureus*, 1 × 10^6^ CFU/mL) was added to 1 mL containing SAC or ZnO dispersions. The mixtures were incubated at 37 °C for 24 h. After incubation, 100 µL aliquots of each culture were spread-plated onto MacConkey’s solid culture medium and bacterial viability per suspension quantified.

### 2.10. Effect of Temperature on the Stability of Single-Atom Zinc Catalysts

SAC and ZnO were separately incubated for 15 min at temperatures of 20 °C, 25 °C, 40 °C, 60 °C, and 80 °C. Samples were suspended in 1 mL LB broth at a concentration of 25 mg/mL. Following this, bacterial suspensions of *S. typhimurium*, *S. aureus*, and *E. coli* (1 mL, 1 × 10^6^ CFU/mL) were added to the solutions. The mixtures were then incubated at 37 °C with shaking for 24 h. Then, the bacterial load in the solution was quantified by the plate count method.

### 2.11. Effect of pH on the Stability of Single-Atom Zinc Catalysts

SAC and ZnO were individually suspended in water with varying pH levels (2.0, 3.0, 4.0, 5.0, 6.0, 7.0, and 8.0) and allowed to stand at 25 °C for 24 h. After this period, the catalysts were recovered and dried in the shade. Samples were then suspended in 1 mL LB broth at a concentration of 25 mg/mL. Bacterial suspensions of *S. typhimurium*, *S. aureus* and *E. coli* (1 mL, 1 × 10^6^ CFU/mL) were added to the solutions, which were then incubated at 37 °C for 24 h. The bacterial concentration was subsequently assessed using the plate count method in the solution.

### 2.12. Animal Experiment

#### 2.12.1. Groups and Treatments

The basic ration consisted of a corn–soybean meal-type diet, adjusted according to the feeding standards for Sanhuang chickens and the nutritional needs outlined by the NRC (1994), as detailed in Table 1.

A total of 200 one-day-old Sanhuang chickens were randomly divided into four groups with five replicates per group and ten chickens per replicate. The groups included the negative group (NC), *S. typhimurium* group (STM), zinc oxide group (ZO + STM), and SAC group (SAC + STM).

Specific operations were as follows: The NC and STM groups were provided with a basal diet. The ZO + STM group was given a basal diet containing 150 mg/kg ZnO. The SAC + STM group was administered a basal diet with 150 mg/kg single-atom zinc catalysts. On the 7th and 8th days, chickens in the STM, ZO + STM, and SAC + STM groups were orally inoculated with 500 μL of *S. typhimurium* (10^9^ CFU/mL) for two consecutive days. On the 14th day, chickens in all experimental groups were euthanized.

#### 2.12.2. Clinical Observations and Weight Monitoring

All chickens were weighed at the beginning and end of the experiment. The weight gain rates were normalized to the NC group (100%). The clinical performance of the chickens in each test group was observed daily throughout the experiment.

#### 2.12.3. Bacterial Load of S. typhimurium in the Organs

Liver, spleen, and cecal tissues (25 g) were homogenized in 225 mL of sterile PBS buffer in EP tubes. Then, 1 mL of homogenate was aspirated for colony enumeration.

#### 2.12.4. Histological Pathology Observation

The chickens were euthanized and dissected to assess damage to the gastrointestinal tract (small intestine, jejunum, and cecum) and other organs. The liver and jejunum were processed into paraffin sections, which were subsequently stained with HE. The height and depth of intestinal villi in the duodenum were measured to evaluate the extent of intestinal damage.

#### 2.12.5. Immune Organ Index

The thymus, spleen, and bursa of Fabricius were then weighed, and the immune organ index was subsequently calculated from the weights. Ten replicates from each group were randomly sampled.

$$\mathrm{Immune}\;\mathrm{organ}\;\mathrm{index}\;\left(\%\right)=\frac{\mathrm{Immune}\;\mathrm{organ}\;\mathrm{weight}\;(g)\;\times \;100\%}{\;\mathrm{Live}\;\mathrm{chicken}\;\mathrm{weight}\;(g)}$$

#### 2.12.6. Quantitative PCR

Ileal tissue (50 mg) was pulverized in liquid nitrogen; total RNA was isolated and retro-transcribed. Receptor expression levels (IL-4, IL-6, IL-10, iNOS, claudin-1, and occludin) were measured using the QuantStudio 7 Flex (Thermo Fisher Scientific, Waltham, MA, USA). The primers listed in Table 2 were utilized.

#### 2.12.7. Intestinal Flora Analysis

Six samples of cecal contents (2 mL/sample) were randomly selected from chickens of each group, and then cryopreserved for gut flora analysis.

### 2.13. Statistical Analysis

All data were analyzed with ANOVA and the t-test method and represented as means ± SD with the software SPSS 22.0 (SPSS Inc., Chicago, IL, USA). *p* value < 0.05 was considered statistically significant. Real-time qPCR data were analyzed by the 2^−ΔΔCt^ method and presented as fold changes in gene expression after normalizing to the internal control β-actin gene expression level.

## 3. Results

### 3.1. Characterization of SAC

**Figure 2:**
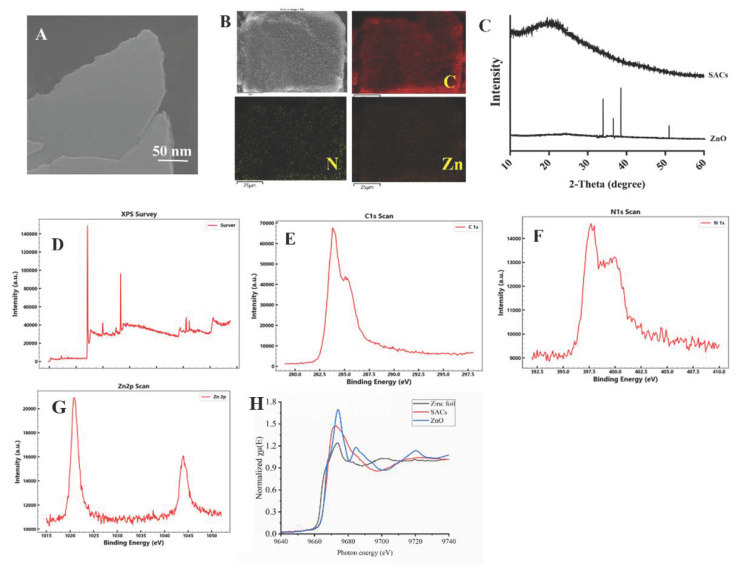
Characterization of the SAC catalyst. (**A**) Field-emission scanning electron microscopy (FE-SEM) image of SAC. (**B**) SEM image and corresponding energy-dispersive X-ray spectroscopy (EDX) elemental mapping of C, N, and Zn. (**C**) X-ray diffraction (XRD) patterns of SAC and ZnO. (**D**) X-ray photoelectron spectroscopy (XPS) survey spectrum of SAC. High-resolution XPS spectra of (**E**) C 1s, (**F**) N 1s, and (**G**) Zn 2p for SAC. (**H**) Zn K-edge X-ray absorption near-edge structure (XANES) spectra of SAC, Zn foil, and ZnO.

The appearance of SAC is shown in Figure 2A. The obtained images exhibited excellent consistency with the microstructural features observed in the FESEM measurements reported by Mu [17]. EDS mapping analysis revealed that the surface of the SAC is enriched with Zn (Figure 2B). Subsequently, XRD patterns revealed no distinct diffraction peaks attributable to ZnO, indicating the absence of granular aggregation in the metallic zinc phase of the zinc-loaded SAC (Figure 2C).

XPS spectra were acquired to elucidate the chemical state of SAC. These spectra confirm the coexistence of carbon (C), nitrogen (N), and zinc (Zn) in the SAC (Figure 2D). In the high-resolution C 1s spectrum, three distinct peaks emerged at binding energies of 285.1, 285.3, and 283.8 eV, assigned to N=C=N, C—NH, and C=C functional groups, respectively (Figure 2E). The N 1s spectra (Figure 2F) displayed five peaks at 399.9, 399.6, 399.2, 398.6, and 397.6 eV, which were assigned to oxidized N, graphitic N, pyrrolic N, Zn–N coordination, and pyridinic N, respectively. Analysis of the Zn 2p spectra (Figure 2G) revealed two peaks at 1020.8 and 1044.0 eV, corresponding to the Zn 2p_3_/_2_ and Zn 2p_1_/_2_ spin–orbit components of Zn^2+^ ions. Figure 2H shows the normalized Zn K-edge XANES spectra of zinc foil, SAC, and ZnO. The absorption edge of SAC is close to that of ZnO, indicating that the Zn species in SAC are primarily in the +2 oxidation state. Moreover, the white-line intensity of SAC at around 9680 eV lies between that of zinc foil and ZnO, suggesting that Zn in SAC is coordinated by N or O atoms with a coordination number lower than that of ZnO. The absence of metallic Zn features further confirms the atomic dispersion of Zn in SAC.

### 3.2. Antibacterial Activity of SAC

The minimum inhibitory concentration (MIC) and minimum bactericidal concentration (MBC) of SAC against *S. typhimurium*, *E. coli*, and *S. aureus* were determined by microdilution of the soup. SAC showed excellent broad-spectral activity at MIC 3.125 mg/mL and MBC 25 mg/mL for all pathogens (Table 3). In particular, SAC was significantly higher than ZnO (MIC: 12.5 mg/mL; MBC: 80 mg/mL). In thermal stability tests, SAC remains fully active for 15 min after exposure at 80 °C, while ZnO loses 85% activity under similar conditions (Table 4). pH resistance testing confirmed that SAC retains > 90% activity in the pH range 2–8 and exceeds ZnO (*p* < 0.05) (Table 5). Dynamic fluctuations in pH suggested that SAC had stronger antimicrobial activity compared to ZnO, making it more resistant to the gastrointestinal tract.

### 3.3. In Vivo Therapeutic Efficacy

#### 3.3.1. Clinical Outcomes and Weight Gain

**Figure 3:**
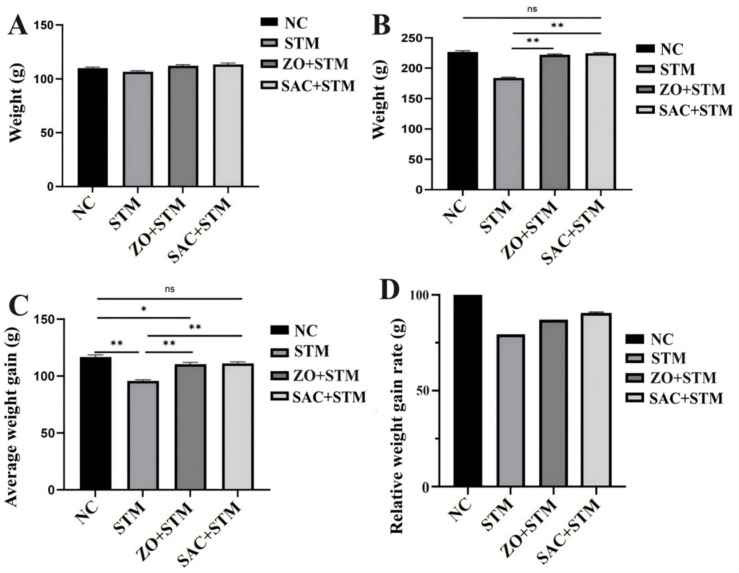
Weight changes in Sanhuang chickens treated with ZO and SAC following infection with *S. typhimurium*. (**A**) Body weight at 0 dpi. (**B**) Body weight at 5 dpi. (**C**) Average weight gain at 1–6 dpi. (**D**) Relative weight gain rate at 1–6 dpi. *n* = 10, * *p* < 0.05, ** *p* < 0.01.

Chickens infected with *S. typhimurium* (STM group) exhibited severe clinical symptoms, including lethargy, anorexia, and diarrhea, leading to a 20.2% reduction in weight gain compared to the NC group (*p* < 0.05). No significant difference was observed in relative weight gain rates between the ZO + STM and SAC + STM groups (Figure 3A–C). No mortality was observed in any group throughout the experiment.

#### 3.3.2. Bacterial Clearance in Target Organs

**Figure 4:**
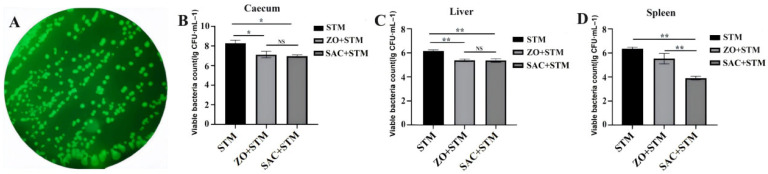
The amount of *S. typhimurium* in the cecum, liver and spleen of Sanhuang chickens at 5 dpi. (**A**) SM022 under a fluorescence microscope. (**B**–**D**) Plate counts were used to determine the amount of SM022 in the cecum, liver and spleen. *n* = 10, * *p* < 0.05, ** *p* < 0.01.

FITC-labeled *S. typhimurium* colonies were examined by fluorescence microscopy (Figure 4A). The bacterial load in the cecum, liver, and spleen tissues of the ZO + STM and SAC + STM groups was lower than that of the STM group (*p* < 0.05). Furthermore, SAC + STM had a significantly lower bacterial load in the spleen than ZO + STM (*p* < 0.05) (Figure 4D).

#### 3.3.3. The Histopathological Observation of the Intestine

**Figure 5:**
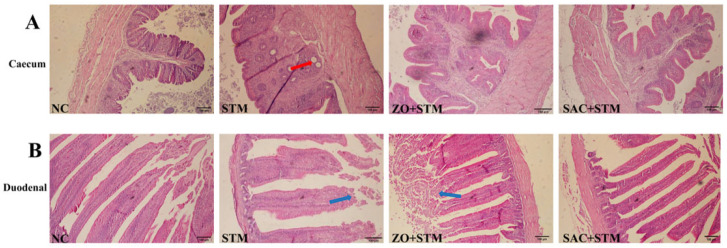
Histological analysis of the cecum and duodenum tonsils. (**A**) Cecum and (**B**) duodenum (100×, HE; scale bar, 100 μm). (The red arrow is epithelial cell necrovascization; the blue arrow indicates significant exposure at the tip of the villi of the small intestine).

NC group cecal mucosa was intact with neatly arranged, plump villi, a clear mucosal–muscular-layer boundary, and no inflammatory cell infiltration, tissue edema, or pathological damage. In contrast, the STM group had typical cecal pathological damage (red arrows), with crypt destruction, villous edema and shortening, and focal epithelial detachment, consistent with Salmonella-induced intestinal inflammation. Compared with the STM group, the ZO + STM group had significantly reduced cecal damage, less inflammatory infiltration, relatively intact villi, and nearly restored crypts, indicating that zinc oxide nanoparticles alleviated Salmonella-induced cecal damage. The SAC + STM group cecal mucosa was similar to the NC group, with neat, intact villi and no inflammatory infiltration or tissue edema (Figure 5A).

NC group duodenal villi were finger-like, neat, and dense, with an intact epithelium, no lamina propria inflammation, and a normal muscular layer. The STM group had severe duodenal damage (blue arrows), with villous atrophy/shortening/lodging, epithelial necrosis, and disordered lumen. Compared with STM, ZO + STM had significantly reduced duodenal damage, partially restored villous length, and less inflammation; mild villous breakage/detachment remained (blue arrows). SAC + STM duodenal villi were intact, neat, and similar to NC, with no inflammation or epithelial damage (Figure 5B).

**Figure 6:**
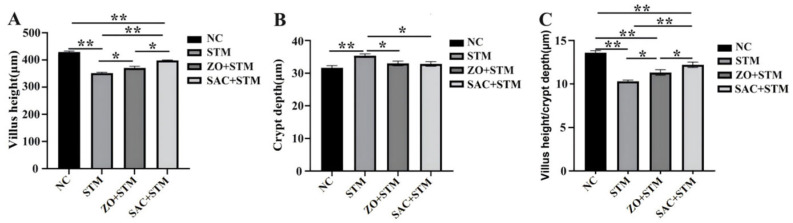
The effect of a single-atom zinc catalyst on the duodenal tissue morphology of *S. typhimurium*-infected Sanhuang chickens. (**A**) Villus height (μm); (**B**) Crypt depth (μm); (**C**) Villus height/crypt depth ratio. *n* = 10, * *p* < 0.05, ** *p* < 0.01.

The SAC + STM group exhibited a 1.52-fold increase (13.12 ± 0.3 vs. 8.61 ± 1.8 in STM group, *p* < 0.001) (Figure 6).

#### 3.3.4. Histopathological Observation of Liver

**Figure 7:**
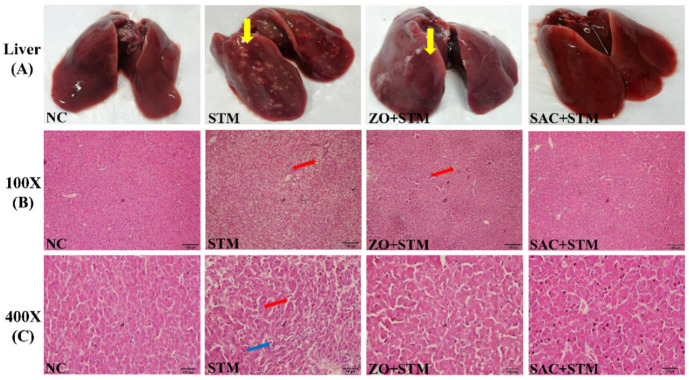
Focal liver lesion. (**A**) The lesions on the surface of the liver (red arrow). (**B**,**C**) Histological analysis of the liver (100×; 400×; HE). (Yellow arrows indicate focal necrotic foci; red arrows indicate areas of hepatocyte edema; and blue arrows indicate inflammatory cell infiltration.)

Observation of livers in the NC group revealed smooth surfaces, uniform color, clear and intact liver parenchyma, and orderly hepatocyte arrangement with the central vein as the boundary. In STM group, livers exhibited numerous gray-white foci, local congestion and swelling, blurred parenchyma structure, vascular stasis, disorderly hepatocyte arrangement, erythrocyte stacking, rupture of hepatocellular membranes, nuclear consolidation, extensive hepatocyte necrosis, and significant inflammatory cell infiltration. In contrast, livers from the SAC + STM and ZO + STM groups displayed only a few sporadic gray-white foci on the surface, and maintained intact parenchyma structures and regular hepatocyte arrangement with mild congestion and limited inflammatory cells and necrosis. The degree of lesions in the ZO + STM group was less severe than in the SAC + STM group (Figure 7).

#### 3.3.5. Immune Organ Index Results

**Figure 8:**
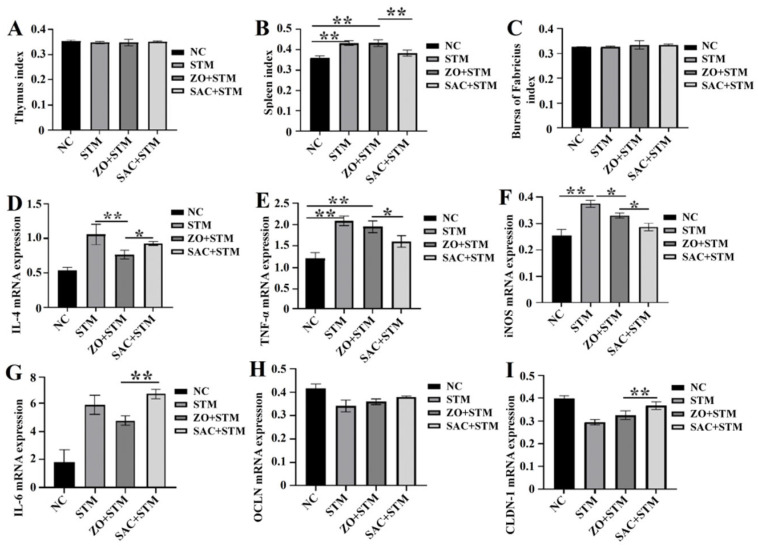
The effects of ZO and SAC on the immune organs and jejunum tissues of chickens infected with *S. typhimurium*: (**A**–**C**) 14 d thymus index, spleen index and bursa of Fabricius index; IL-4 (**D**), TNF-α (**E**), iNOS (**F**), IL-6 (**G**), OCLN (**H**) and claudin-l (**I**) mRNA expression levels in the jejunum were determined. *n* = 10 * *p* < 0.05, ** *p* < 0.01.

There were no significant differences in the thymus and bursa of Fabricius indices among the treatment groups compared to the NC group (*p* > 0.05). However, spleen indices of the SAC + STM group were lower than the ZO + STM group (*p* < 0.05) (Figure 8B).

#### 3.3.6. Expression of mRNA

The effects of single-atom zinc on mRNA expression in the ileum of infected Sanhuang chicken are shown in Figure 8D–I. Compared to the NC group, the STM group exhibited significantly higher levels of pro-inflammatory factors IL-6 and iNOS as well as claudin-1 mRNAs. These findings indicate that *S. typhimurium* infection in the STM group disrupted intestinal structure, triggering immune activation and severe inflammatory responses. In contrast, broilers in the SAC + STM group showed markedly reduced levels of inflammatory factors (TNF-α and iNOS) compared to the ZO + STM group (*p* < 0.05).

#### 3.3.7. Sequencing Sample Data and Clustering Analysis of ASVs in the Cecum

**Figure 9:**
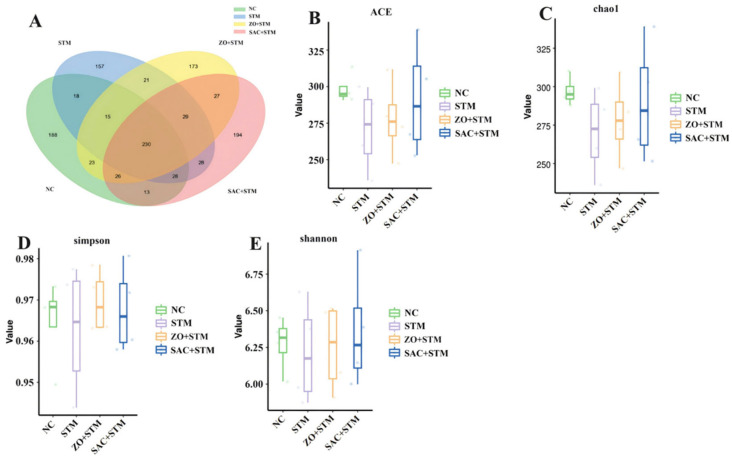
SAC affect the bacterial composition of the gut microbiota. (**A**) Cluster analysis of ASVs. (**B**) Shannon–Wiener curve. (**C**) Coverage analysis of Good’s sequencing data. (**D**) Dilution curve. (**E**) Species accumulation curve.

The number of species shared among the NC, STM, ZO + STM, and SAC + STM groups was 230. The NC group had 541 species, STM group had 526 species, ZO + STM group had 544 species, and SAC + STM group had 575 species. The highest fecal microbiota composition was observed in the SAC + STM group and the lowest in the STM group, although the difference was not significant (Figure 9A).

#### 3.3.8. Analysis of Microbial α-Diversity in the Cecum

The ACE index (Figure 9B), Chao1 index (Figure 9C), and observed species index (Figure 9D) were used to evaluate microbial richness, while the Shannon index (Figure 9E) assessed species diversity. Although there were no statistically significant differences in alpha-diversity indices among the groups, the SAC + STM group exhibited higher ACE, Chao1, and Shannon indices compared to the NC, STM, and ZO + STM groups, indicating a potential trend toward improved microbial richness and diversity.

#### 3.3.9. Structure and Distribution of Microbial Populations in the Cecum

**Figure 10:**
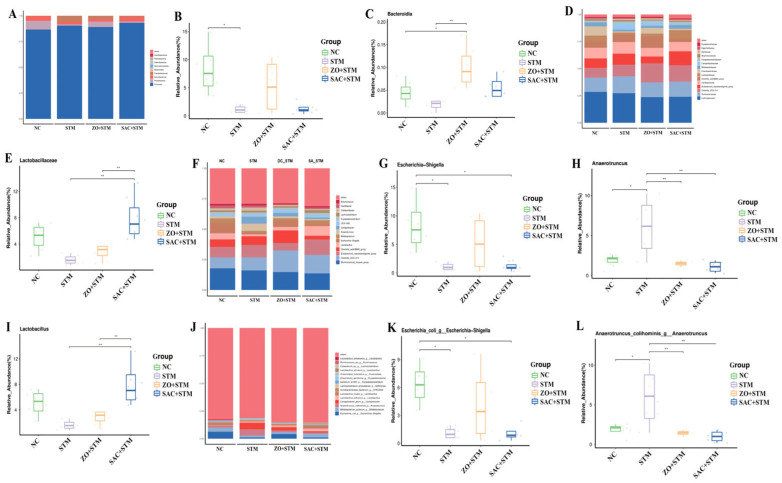
Effect of SAC on the cecal microflora of Sanhuang chickens. The difference in relative abundance at the phylum (**A**), family (**D**), genus (**F**) and species (**J**) levels. Proteobacteria (**B**) and Bacteroidota (**C**) differ in phylum abundance at the phylum level. *Lactobacillus* (**E**) differs in family abundance at the genus level. *Escherichia-Shigella* (**G**), *Anaerotruncus* (**H**) and *Lactobacillus* (**I**) differ in genus abundance at the genus level. *Escherichia_coli_g Escherichia-Shigell* (**K**) and *Anaerotruncus_colihominis_g Anaerotruncus* (**L**) differ in species abundance at the genus level. *n* = 6, * *p* < 0.05, ** *p* < 0.01.

Microbial population in the cecum of Sanhuang chickens was predominantly composed of *Firmicutes*, *Proteobacteria*, *Actinobacteriota*, *Campilobacterota*, and *Bacteroidota* (Figure 10A). *Firmicutes* had the highest relative abundance, with 86.86%, 90.36%, 89.38%, and 93.23% in the NC, STM, ZO + STM, and SAC + STM groups, respectively. The number of *Proteobacteria* was significantly lower (*p* < 0.05) in the STM and SAC + STM groups compared to the NC group (Figure 10B), while the relative abundance of *Bacteroidota* was significantly higher in the ZO + STM group (Figure 10C).

At the family level, the microbial population of the chick cecum included *Lachnospiraceae*, *Ruminococcaceae*, *Enterobacteriaceae*, *Clostridia_vadinBB60_group*, *Oscillospiraceae*, and *Lactobacillaceae* (Figure 10D). *Lachnospiraceae* had the highest relative abundance, with 33.72%, 40.18%, 25.84%, and 25.26% in the NC, STM, ZO + STM, and SAC + STM groups, respectively. The relative abundance of *Lactobacillaceae* was significantly increased (*p* < 0.05) in the SAC + STM group compared to the STM group (Figure 10E).

At the genus level, the microbial populations were primarily composed of *Ruminococcus* (*[Ruminococcus]_torques_group*), *Clostridium* (*Clostridia_UCG-014*), *Eubacterium* (*[Eubacterium]_coprostanoligenes_group*), *Escherichia-Shigella*, *Anaerotruncus*, *Bifidobacterium*, and *Lactobacillus* (Figure 10F). The relative abundance of *Escherichia-Shigella* was significantly lower (*p* < 0.05) in the STM and SAC + STM groups compared to the NC group (Figure 10G). The relative abundance of *Anaerotruncus* was significantly lower (*p* < 0.05) in the NC, ZO + STM, and SAC + STM groups compared to the STM group (Figure 10H), indicating that *S. typhimurium* infection significantly increased the relative abundance of *Anaerotruncus* (Figure 10I). The relative abundance of *Lactobacillus* was significantly higher in the SAC + STM group compared to the STM and ZO + STM groups, suggesting that the addition of single-atom zinc catalysts to the diets significantly increased the relative abundance of *Lactobacillus*.

At the species level, the dominant flora of the chick gut consisted mainly of *Escherichia coli-Shigella (Escherichia_coli_g Escherichia-Shigella), Bifidobacterium pullorum (g Bifidobacterium), Campylobacter jejuni (g Campylobacter), Anaerotruncus colihominis (g Anaerotruncus), Bifidobacterium*, and *Lactobacillus salivarius (g Lactobacillus)*, among others (Figure 10J). The relative abundance of *Escherichia coli g Escherichia-Shigella* was significantly lower (*p* < 0.05) in the STM and SAC + STM groups compared to the NC group (Figure 10K). The relative abundance of *Anaerotruncus_colihominis g Anaerotruncus* was significantly lower (*p* < 0.05) in the NC, ZO + STM, and SAC + STM groups compared to the STM group (Figure 10L), further indicating that *S. typhimurium* infection significantly increased the relative abundance of *Anaerotruncus* (*p* < 0.05).

## 4. Discussion

Single-atom zinc catalysts (SAC), an innovative nutritional intervention strategy, have demonstrated multiple significant advantages and broad application potential in ensuring animal-derived food safety.

### 4.1. High Antibacterial Activity: Effective Inhibition of Foodborne Pathogens at Low Doses

In this study, MIC and MBC of both ZnO and SAC against *S. typhimurium*, *E. coli*, and *S. aureus* were systematically determined. The results revealed that SAC exhibited four-fold greater antimicrobial activity than ZnO while requiring a significantly lower zinc dose (Table 3 and Table 6). This study demonstrated the potential of single-atom zinc materials in synergistically inhibiting multiple foodborne pathogens (*S. typhimurium*, *E. coli*, and *S. aureus*), filling the application gap in multi-pathogen control for this material system.

The fundamental advantage of SAC lies in their exceptionally high atomic utilization efficiency. Compared to conventional ZnO, the zinc in SAC exists as atomically dispersed active sites, achieving nearly 100% bioavailability. This property translates directly into two key application advantages—(1) high efficacy: SAC exhibits broad-spectrum and potent inhibition against *S. typhimurium*, *E. coli*, and *S. aureus*; (2) low loading: zinc addition is significantly reduced to achieve equal or superior protection, thereby minimizing zinc migration along the food chain and environmental accumulation at the source.

Furthermore, in vivo validation using the three-yellow-chicken model corroborated these findings. As detailed in Table 6, SAC demonstrated superior pathogen clearance efficacy compared to conventional ZnO. Specifically, while both treatments reduced cecal bacterial load, SAC achieved a significantly lower splenic load (*p* < 0.05), indicating a more effective blockade of systemic dissemination—a critical advantage for preventing foodborne illness at the source.

Further in vivo experiments demonstrated that the *S. typhimurium* loads in the cecum, liver, and spleen of infected chicks were significantly reduced by SAC (*p* < 0.05), with superior efficacy compared to the ZnO-treated group (Figure 4). These results highlight the direct application value of SAC in controlling *S. typhimurium* contamination in poultry products and enhancing food safety.

### 4.2. Exceptional Stability: A Delivery Platform Adapted to Complex Food Chain Environments

The stability of SAC is crucial for their efficacy as food safety control agents. Zn species in SAC are primarily in the +2 oxidation state; experiments demonstrated that SAC retained 100% activity after treatment at 80 °C for 15 min, whereas ZnO lost 85% of its activity (Table 4). Furthermore, SAC maintained over 90% activity across a broad pH range (2–8), demonstrating significantly greater acid–base tolerance than the pH-sensitive ZnO (Table 5). This may be due to the fact that the metal atoms in ZnO are mainly attracted to each other by van der Waals forces or metal bonds, and are prone to migration, aggregation and even sintering at high temperatures. SAC anchor a single Zn atom to the surface of MOF through coordination bonds and lack of direct contact between atoms, fundamentally avoiding agglomeration, thus having stronger thermal stability and acid resistance. These thermal stability and acid tolerance properties enable SAC to withstand harsh conditions during food processing (e.g., high-temperature sterilization), storage, and transit through the animal gastrointestinal tract, ensuring the effective delivery of active components to target sites such as the intestines.

In contrast, the bioavailability of conventional ZnO is limited because only surface zinc atoms are accessible for interaction in the gut, a constraint imposed by its crystalline structure. SAC overcome this limitation through a single-atom anchoring technique, which maximizes the exposure of active zinc sites on the carrier surface, achieving an atomic utilization rate approaching 100%. This high-efficiency design reduces the environmental residue risk associated with zinc over-supplementation and enhances the control efficacy per unit dose. These attributes align with the green development goals in food safety, which emphasize reduced input and enhanced efficiency.

### 4.3. Protective Effects: From Pathogen Clearance to Microbiota Remodeling

The potential of SAC extends beyond direct bactericidal activity to encompass systemic protection of host health. Chicks, with their underdeveloped immune systems, are particularly vulnerable to *S. typhimurium* infections, which result in symptoms such as diarrhea, organ enlargement, and intestinal hemorrhage, ultimately impairing their feeding efficiency and growth rates demonstrated that high-zinc diets significantly improved the growth performance of broilers infected with *S. typhimurium*. By employing an *S. typhimurium*-infected chick model [18,19,20], we demonstrated that SAC support animal health and product quality through a tripartite mechanism.

#### 4.3.1. Inhibition of Pathogen Adhesion and Invasion

Adhesion and invasion of pathogens were inhibited by SAC, reducing *S. typhimurium* colonization in organs (liver, spleen, etc.) (Figure 4B–D) and alleviating liver injury severity (Figure 7). Anderson [21] confirmed that zinc infusion following intraperitoneal injection of *S. typhimurium* endotoxin prevented liver necrosis and mortality.

#### 4.3.2. Alleviation of Intestinal Inflammation

The intestinal tract, the largest immune organ in animal hosts, harbors a rich population of immune cells. A regulatory network is formed by these immune cells through cytokine secretion—one that not only regulates the activation and responses of diverse immune cell types but also interacts with systemic immune responses [22,23]. Analysis of intestinal cytokines in our model confirmed the role of this pathway in *S. typhimurium* infection. Specifically, compared to the ZO + STM group, chicks treated with SAC (SAC + STM group) exhibited a significant downregulation of key pro-inflammatory cytokines, including iNOS and TNF-α (*p* < 0.05; Figure 8D–G). This reduction in inflammation was associated with structural and functional improvements in the gut. SAC promoted repair of the intestinal mucosa, as evidenced by a significant increase in villus height (*p* < 0.05), which contributed to the maintenance of normal absorptive function. Consequently, SAC helped prevent the growth retardation typically caused by infection-induced diarrhea and reduced feed intake (Figure 5). Additionally, Chang et al. found that under 70 mg/kg of zinc–glycine (Zn-Gly) was more effective than an equivalent dose of ZnSO_4_ in regulating gut morphology [15]. Significant differences exist in the regulatory effects of zinc formulations on intestinal function, with the intensity of action varying by formulation.

#### 4.3.3. Optimization of Intestinal Structure

The impairment of intestinal function by *S. typhimurium* infection is primarily mediated through two interrelated pathological mechanisms. The first mechanism involves damage to the intestinal physical barrier and mucosal integrity. The pathogen first targets tight junctions between intestinal epithelial cells, disrupting protein complexes (e.g., occludin, claudin-1) via toxic factor secretion, leading to loss of mucosal integrity [24] and subsequent breach of gut continuity that facilitates the translocation of luminal harmful substances [25]. Notably, zinc has been shown to protect barrier function by promoting tight junction protein expression to reduce intestinal fluid leakage [26]. The second mechanism involves direct invasion of intestinal epithelial cells and microvillus damage. After breaching the barrier, bacteria invade epithelial cells and release toxins that damage microvilli (key structures for absorption), resulting in shortened, atrophied, and less dense villi and significantly impaired nutrient absorption [27]. These two mechanisms act synergistically to induce clinical symptoms such as diarrhea and electrolyte imbalance, ultimately compromising host health.

In terms of intestinal structure optimization, the SAC + STM group increased the villus height/crypt depth (VH/CD) ratio and upregulated claudin-1 mRNA expression compared to the ZO + STM group (*p* < 0.05, Figure 6 and Figure 8I). These results indicate that SAC help maintain intestinal mucosal integrity during *Salmonella* challenge by improving gut morphology and enhancing tight junction protein expression. Ma found that dietary zinc supplementation alleviates S. typhimurium-induced intestinal barrier dysfunction, partly via increased occludin and claudin-1 expression in broilers [28]. Leveraging the high bioavailability of single-atom zinc, SAC promote tight junction protein expression more efficiently than conventional ZnO, whose action is limited to surface zinc atoms. This enhanced efficacy strengthens the intestinal epithelial barrier, leading to superior preservation of intestinal structure and function, thereby providing a structural basis for protection against *Salmonella*-induced enteric damage.

#### 4.3.4. Modulation of Gut Microbiota

SAC modulated the intestinal microecology, increasing the abundance of beneficial bacteria (e.g., *Lactobacillus*; 25.2%) compared to the ZnO group (14.7%) and reducing the proportion of opportunistic pathogens (e.g., *Proteobacteria*; 28.7% vs. 12.4% in the ZnO group). SAC also restored the *Firmicutes*/*Bacteroidetes* ratio (0.62 vs. 2.34). These differences highlight the advantage of SAC in selectively reshaping the microbial community. This finding is consistent with Wang [29], who reported that dietary supplementation with 60 mg/kg zinc glycinate increased the abundance of *Lactobacillus*. Furthermore, dietary zinc intervention has been observed to alter gut microbiota composition and metabolite profiles in mice [30], with both zinc deficiency and excess inducing microbial dysbiosis. By reconstructing this intestinal biological barrier, SAC exert a synergistic “bactericidal–anti-inflammatory–microbiota-modulating” effect. This multi-pronged approach reduces the risk of *Salmonella* contaminating the environment via feces or entering the food chain at the source, thereby enhancing the safety of poultry products.

In summary, an innovative solution for controlling *Salmonella* contamination in poultry products is offered by SAC through their comprehensive advantages of high antibacterial efficacy, stable delivery, and ecological regulation. These characteristics—high efficiency at low doses and environmental friendliness—align with the dual demands of food safety and sustainable development, and SAC are positioned as a promising core carrier for next-generation food safety control technologies [31].

## 5. Conclusions

This study presents a MOF-based single-atom zinc catalyst (SAC), which represents the first documented application of a single-atom catalyst in animal feed. The core innovation resides in the SAC architecture—atomically dispersed active sites anchored on a carrier—which achieves approximately 100% atomic utilization efficiency, thereby distinguishing it from conventional bulk zinc supplements.

Comprehensive in vivo and in vitro evaluations demonstrate that SAC serves as a highly effective and environmentally favorable alternative to conventional zinc oxide (ZnO) in poultry nutrition. Compared to established supplements, SAC demonstrates a synergistic integration of potent antibacterial activity, targeted modulation of the gut microbiota, and systemic anti-inflammatory effects. Mechanistically, SAC effectively eradicates Salmonella infections while restoring intestinal barrier integrity and reshaping the gut microbiome toward a healthier profile.

A key practical implication of this high-efficiency design is the potential for significantly reduced zinc dosage to achieve superior protective efficacy, thereby mitigating environmental zinc accumulation and food chain migration. While direct dosage reduction metrics require further quantification, these findings establish a viable direction for the industrial application of single-atom materials in livestock sustainability. Future validation in large-scale field conditions will be essential to confirm the robustness and long-term efficacy of this feeding strategy.

## Figures and Tables

**Table 1:** Composition of basal diets (air-dry basis) (%).

Items	1 to 14 Days
Dietary composition	
Corn	55.00
Soybean meal	37.20
Soybean oil	4.45
Limestone	1.15
NaCl	0.25
CaHPO_4_	1.95
Total	100.00
Nutrient levels	
Metabolizable energy (MJ/kg)	12.45
Crude protein	21.15
Ca	0.92
P	0.65
Lysine	1.18
Methionine	0.47

**Table 2:** Primers used for qRT–PCR.

Gene Name	Sequences (5′ to 3′)
*β-actin*	F: CCCTGTATGCCTCTGGTC
	R: CTCGGCTGTGGTGGTGAA
*TNF-α*	F: TGTCTCAGAATGAGGCTGGATAA
	R: TCAGGGAAGAATCTGGAAAGGT
*IL-4*	F: TCCCTCAAGGTAAGGCTCTGT
	R: GGCTGTGTGAGAGGAGAACG
*IL-6*	F: AGGACGAGATGTGCAAGAAGTTC
	R: TTGGGCAGGTTGAGGTTGTT
*iNOS*	F: TCCTGTGACATGACACCCAGAGAG
	R: AAGCACGGGACTGTTTCATTGAGAG
*Claudin-l*	F: ATGACCAGGTGAAGAAGATGC
	R: TGCCCAGCCAATGAAGAG
*OCLN*	F: TGTCTGTGGGTTCCTCATCG
	R: TTCTTCACCCACTCCTCCACG

Note: *OCLN*—*occludin* gene.

**Table 3:** Bacteriostatic effect of a single-atom zinc catalyst at different concentrations.

Group	Concentration (mg/mL)	SM022 (CFU/mL)	*E. coil* (CFU/mL)	*S. aureus* (CFU/mL)
Negative control group	0	9.93 × 10^8^	7.19 × 10^8^	6.69 × 10^8^
SAC group	100	0	0	0
	80	0	0	0
	50	0	0	0
	40	0	0	0
	25	3.48 × 10^2^	4.06 × 10^2^	9.74 × 10^2^
	20	3.19 × 10^2^	8.24 × 10^3^	6.52 × 10^4^
	12.5	7.85 × 10^3^	6.83 × 10^4^	1.00 × 10^5^
	6.25	9.32 × 10^6^	1.75 × 10^6^	1.35 × 10^7^
	3.125	3.58 × 10^7^	1.39 × 10^7^	2.08 × 10^7^
	1.5625	5.97 × 10^7^	7.15 × 10^7^	5.08 × 10^7^

Note: SM022, *S. typhimurium*; *E. coli*, *Escherichia coli*; *S. aureus*, *Staphylococcus aureus*.

**Table 4:** Effects of different temperatures on the bacteriostatic effects of SAC.

Groups	Temperature (°C)	SM022 (CFU/mL)	*E. coil* (CFU/mL)	*S. aureus* (CFU/mL)
Negative control group	25	4.6 × 10^8^	5.23 × 10^8^	5.65 × 10^8^
SAC group	20	3.7 × 10^2^	1.38 × 10^2^	4.38 × 10^2^
	25	4.15 × 10^2^	5.42 × 10^2^	4.67 × 10^2^
	40	3.49 × 10^2^	2.44 × 10^2^	3.72 × 10^2^
	60	3.22 × 10^2^	1.79 × 10^2^	3.82 × 10^2^
	80	2.35 × 10^2^	1.45 × 10^2^	4.28 × 10^2^

Note: SM022, *S. typhimurium*; *E. coli*, *Escherichia coli*; *S. aureus*, *Staphylococcus aureus*.

**Table 5:** Effect of different pH values on the antibacterial effect of a single-atom zinc catalyst.

Groups	PH	SM022 (CFU/mL)	*E. coil* (CFU/mL)	*S. aureus* (CFU/mL)
Negative control group	7	1.79 × 10^8^	1.625 × 10^8^	1.15 × 10^8^
SAC group	2	8.8 × 10^2^	7.84 × 10^2^	8.835 × 10^2^
	3	7.59 × 10^2^	8.77 × 10^2^	8.4 × 10^2^
	4	7.53 × 10^2^	8.75 × 10^2^	7.284 × 10^2^
	5	8.56 × 10^2^	8.947 × 10^2^	8.793 × 10^2^
	6	8.86 × 10^2^	9.013 × 10^2^	8.96 × 10^2^
	7	8.43 × 10^2^	8.787 × 10^2^	8.977 × 10^2^
	8	7.74 × 10^2^	8.7 × 10^2^	7 × 10^2^

Note: SM022, *S. typhimurium*; *E. coli*, *Escherichia coli*; *S. aureus*, *Staphylococcus aureus*.

**Table 6:** Comparison of multi-dimensional core experimental results of SAC and ZnO on Salmonella attack three-yellow-chicken model.

	NC	STM	ZO + STM	SAC + STM
Clinical symptoms	Normal weight gain without clinical symptoms	Compared with the NC group, the weight gain was reduced by 20.2% (*p* < 0.05), and clinical symptoms such as anorexia and diarrhea appeared	No clinical symptoms	No clinical symptoms
Organ carrier capacity	No Salmonella colonization	The bacterial load of the cecum, liver and spleen was significantly higher than that of ZO + STM and SAC + STM (*p* < 0.05)	The bacterial load was significantly lower than that of the STM group (*p* < 0.05), but the spleen load was higher than that of the SAC + STM group *(p* < 0.05)	The bacterial load was significantly lower than that of the STM group (*p* < 0.05), and the spleen load was significantly lower than that of the ZO + STM group (*p* < 0.05)
Pathological and morphological observation of intestinal tissue	Cecum: mucous membrane intact, villi neat and full, no inflammation; duodenum: the villi are arranged in a finger-like fashion, and the epithelium is intact and free of inflammation	Cecum: crypt destruction, villous edema shortening, epithelial shedding (red arrow); duodenum: villous atrophy and necrosis, intestinal lumen disorder (blue arrow)	Cecum: the damage is reduced, the villi are relatively intact; duodenum: length of villi partially restored, mild fracture still seen (blue arrow)	Cecum: structure close to the NC group, no inflammatory edema; duodenum: the villi are intact and undamaged
Immune organ index	Thymus, bursa and spleen indices are normal	Significantly elevated spleen index (inflammatory response)	The spleen index was higher than that of the SAC + STM group (*p* < 0.05)	Spleen index was lower than ZO + STM *(p* < 0.05)
mRNA expression levels of genes in ileal tissue	The basal expression levels of IL-4, IL-6, iNOS, claudin-1, and other markers were measured as described above	Pro-inflammatory factors (IL-6, iNOS, TNF-α) were significantly upregulated, and claudin-1 (tight junction protein) was downregulated	IL IL-4 and iNOS levels were lower than those in the STM group *(p* < 0.05)	The expression levels of TNF-α and iNOS mRNA were significantly lower than those in the ZO + STM group (*p* < 0.05), while the expression of claudin-1 was significantly higher than that in ZO + STM (*p* < 0.05)
Gut microbiota analysis	The number of species is 541, and the dominant bacteria are Firmicutes and *Lactobacillus*	A total of 526 species were identified; Proteobacteria was significantly increased (*p* < 0.05), along with elevated levels of Escherichia-Shigella	The number of species was 544, Bacteroidota was significantly increased (*p* < 0.05), and Lactobacillus was lower than that in the SAC + STM group	The number of species was 575 (the highest), *Lactobacillus* spp. were significantly increased (25.2% vs. ZO + STM group 14.7%, *p* < 0.05), and *E. coli*-*Shigella* spp. were significantly decreased (*p* < 0.05)

## Data Availability

The original contributions presented in this study are included in the article. Further inquiries can be directed to the corresponding authors.

## Abbreviations

ZnO	Zinc oxide
BMOFs	Bio-metal–organic frameworks
Zn-NCs	Zinc nanoclusters
PBS	Phosphate-buffered saline
HE	Hematoxylin–eosin
CFU	Colony-forming units
VH/CD	Villus height/crypt depth ratio
